# The potential for mitochondrial therapeutics in the treatment of primary open-angle glaucoma: a review 

**DOI:** 10.3389/fphys.2023.1184060

**Published:** 2023-08-02

**Authors:** Grace Kuang, Mina Halimitabrizi, Amy-Ann Edziah, Rebecca Salowe, Joan M. O’Brien

**Affiliations:** ^1^ Perelman School of Medicine, Scheie Eye Institute, University of Pennsylvania, Philadelphia, PA, United States; ^2^ Penn Medicine Center for Genetics in Complex Diseases, University of Pennsylvania, Philadelphia, PA, United States

**Keywords:** glaucoma, mitochondrial dysfunction, mitochondrial therapeutics, neurodegeneration, oxidative Stress

## Abstract

Glaucoma, an age-related neurodegenerative disease, is characterized by the death of retinal ganglion cells (RGCs) and the corresponding loss of visual fields. This disease is the leading cause of irreversible blindness worldwide, making early diagnosis and effective treatment paramount. The pathophysiology of primary open-angle glaucoma (POAG), the most common form of the disease, remains poorly understood. Current available treatments, which target elevated intraocular pressure (IOP), are not effective at slowing disease progression in approximately 30% of patients. There is a great need to identify and study treatment options that target other disease mechanisms and aid in neuroprotection for POAG. Increasingly, the role of mitochondrial injury in the development of POAG has become an emphasized area of research interest. Disruption in the function of mitochondria has been linked to problems with neurodevelopment and systemic diseases. Recent studies have shown an association between RGC death and damage to the cells’ mitochondria. In particular, oxidative stress and disrupted oxidative phosphorylation dynamics have been linked to increased susceptibility of RGC mitochondria to secondary mechanical injury. Several mitochondria-targeted treatments for POAG have been suggested, including physical exercise, diet and nutrition, antioxidant supplementation, stem cell therapy, hypoxia exposure, gene therapy, mitochondrial transplantation, and light therapy. Studies have shown that mitochondrial therapeutics may have the potential to slow the progression of POAG by protecting against mitochondrial decline associated with age, genetic susceptibility, and other pathology. Further, these therapeutics may potentially target already present neuronal damage and symptom manifestations. In this review, the authors outline potential mitochondria-targeted treatment strategies and discuss their utility for use in POAG.

## 1 Introduction

Primary open-angle glaucoma (POAG) is a chronic ophthalmic disease characterized by progressive retinal ganglion cell (RGC) damage with gradual loss of peripheral-to-central vision. Classically, the etiology of POAG has been primarily attributed to elevated intraocular pressure (IOP) within the anterior chamber of the globe, exerting deleterious mechanical pressure on the optic nerve and surrounding structures. Thus, IOP-lowering therapies, including topical drops, laser, and surgery, are currently the primary treatments for POAG. While they cannot restore vision loss, IOP-lowering therapies can help to slow disease progression and prevent further damage. However, only 30% of patients on IOP-lowering medications experience a decrease in IOP lasting a year (Jampel, 2014). Furthermore, IOP-lowering therapies are ineffective in approximately 30% of patients with POAG, pointing to the influence of other etiologies in disease manifestation (Doucette, 2015). Multiple supplementary mechanisms for glaucomatous optic nerve head (ONH) insult have been suggested: neurotoxin build-up, vascular dysregulation, abnormal glial response, axonal transport disruption, genetic vulnerability, cerebrospinal fluid circulatory failure, mitochondrial dysfunction, and others (Mancino, 2018). New research focus surrounding the vascular theory of glaucoma has investigated the ischemic and reperfusion dysfunction relating to glaucomatous optic neuropathy. Mitochondrial dysfunction has been implicated in vascular endothelial damage, and ischemic injury in turn can produce mitochondrial dysfunction (Qu, 2022). Although the underlying etiology of POAG is still undefined, research evidence proves a multifactorial etiology to the pathology of glaucomatous optic neuropathy. Current therapeutics are often limited and ineffective to target this unclear multifactorial etiology. Thus, there is a great need to identify and study alternative pathogenic mechanisms and treatment options that carry neuroprotective properties. In particular, mitochondrial therapeutics represent a promising area to provide more accessible and effective treatments for POAG (Jampel, 2014; Gorman, 2016; Osborne, 2016).

Mitochondria are integral organelles that affect many cellular functions and are crucial to cell survival. Disruption in mitochondrial function and structure has been linked to an array of diseases, which are generally classified as primary mitochondrial diseases (caused by mitochondrial dysfunction directly) or secondary mitochondrial diseases (caused indirectly) (Murphy and Hartley, 2018). Both primary and secondary mitochondrial diseases include ophthalmic pathologies. A high concentration of mitochondria is present in the eyes, which is a possible explanation for why they are highly affected by and more sensitive to dysregulation in mitochondrial function (Schrier and Falk, 2011; Osborne et al., 2016). In particular, the retina is the most metabolically active tissue in the human body, and RGCs have long axons that contain a high density of mitochondria to generate energy for distal transport (Barron, 2004). Consistent with the observation that mitochondrial diseases preferentially affect tissues with greater energy demands, disruptions in mitochondrial function have been suggested to have a role in the development of POAG (Beidoe and Mousa, 2012; Yang, 2013; Zinovkin and Zamyatnin, 2019).

Evidence suggests that mitochondrial alterations in RGCs are among the first changes to occur in glaucoma, inducing early neuronal alterations that precede neurodegeneration (Williams et al., 2017). Various mechanisms of mitochondrial injury have been proposed, including pathway dysfunction, signaling dysregulation, genetic variations, and the build-up of reactive oxygen species (ROS) caused by hindered or inefficient oxidative phosphorylation (OXPHOS) (Chrysostomou et al., 2013; Williams et al., 2017). Genetic connections between mitochondrial function and POAG have also been discovered in both mitochondrial and nuclear DNA. For example, the expression of *OPA1*, a gene expressed in RGC soma and axons and associated with spontaneous and inherited mitochondrial optic neuropathies, is significantly downregulated in POAG (Yang, 2013). Published findings point to mitochondrial alterations as part of the disease’s etiology and a consequence of cellular degeneration, linking glaucoma to possible primary and secondary mitochondrial diseases (Lee, 2011).

Current mitochondrial therapies under consideration for POAG span a wide degree of interventions, from lifestyle changes to biologic alterations. Despite this variety, these therapeutic options have all demonstrated some biologic benefit applicable to POAG pathophysiology. Main intervention aims include decreasing optic nerve degeneration, cellular oxidative stress, and age-related vulnerability, as well as increasing mitochondrial biogenesis and functioning capacity. Overall goals for mitochondrial therapy research and application include preventing POAG, slowing progression, and reversing disease pathology.

Although researchers are still in the process of understanding mitochondrial pathophysiology, different approaches to mitochondrial treatment have been suggested as treatments for various diseases, including POAG. This paper aims to bring attention to therapies that target mitochondrial dysfunction in POAG, including physical exercise, diet and nutrition, antioxidant supplementation, stem cell therapy, hypoxia exposure, gene therapy, mitochondrial transplantation, and light therapy. Rather than providing an all-encompassing review of the published literature, the authors seek to offer an informative background and overview of the present options and future possibilities of mitochondrial therapeutics in POAG.Review

## 2 Therapeutic approaches

**FIGURE 1:**
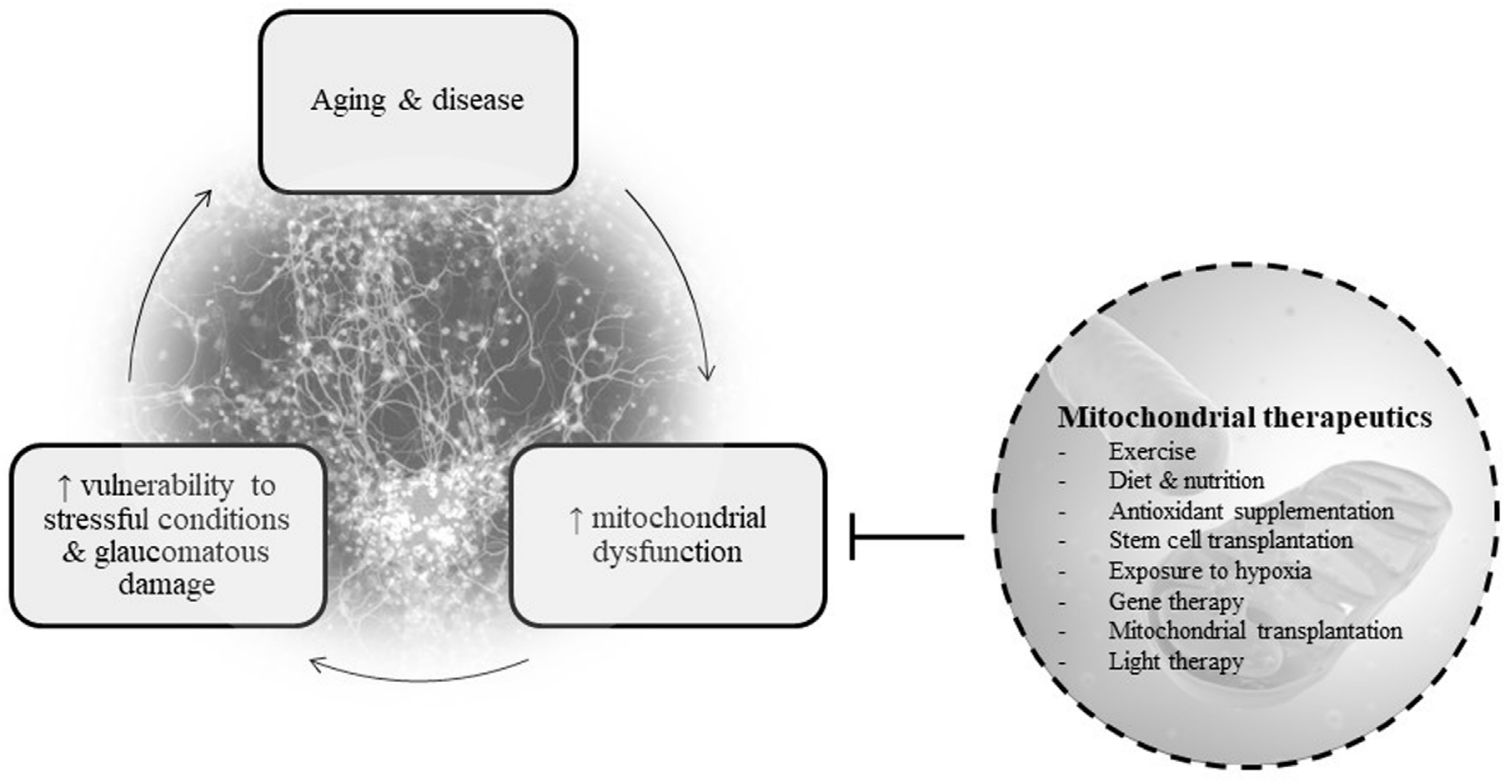
Basic diagram of the relationship between mitochondrial dysfunction, disease, and therapeutics.

Below, we provide an overview of mitochondrial therapeutics for POAG, including key points in each strategy’s mechanism of action, strengths, and limitations. This information is also summarized in Table 1. Figure 1 depicts the basic concept of the vicious cycle of mitochondrial dysfunction and disease (Woods, 2014; Johnson, 2017).

**TABLE 1:** Summary of mitochondria-targeted therapeutic strategies for primary open-angle glaucoma.

Therapeutic strategy	Mechanism of action	Strengths	Limitations
*Exercise*	- Increased physical activity reduces reactive oxygen species (ROS), promotes enzymatic activity for cellular respiration, increases mitochondrial biogenesis and mitophagy, and enhances neurotrophic factors and cerebral pathways	- Low cost	- Greater efficacy studies needed to confirm neuroprotection in glaucoma-
		- Accessible	Lack of clinical trials
		- Non-invasive	- Unclear standardization of recommendations and consistent delivery of treatment
		- Protective against other risk factors such as diabetes	
*Diet and nutrition*	- Altering diet and food intake (ketone-based diet, low-fat diet, Mediterranean diet, vitamin supplementation) or adjusting quantity of caloric intake	- Low cost	- Greater efficacy studies needed to confirm neuroprotection in glaucoma
		- Accessible	- Lack of clinical trials
	- Improves abnormal protein accumulation, neurotoxicity, energy utilization, inflammation, ROS production, and overall mitochondrial dysfunction	- Non-invasive	- Difficult to adhere to in real-world scenarios
		- Protective against other risk factors such as diabetes	- Strict diets can cause inadequate nutrition balances
*Antioxidant supplementation*	- Enzymatic or non-enzymatic antioxidants to counter oxidative stress	- Low cost	- Difficult to achieve specific and concentrated subcellular delivery into mitochondrial organelle
	- Decreased proinflammatory cytokines in the retina and optic nerve	- Accessible	- Improved standardized methods of effective antioxidant supplementation and augmentation strategies needed
	- Increased retinal ganglion cell (RGC) survival and axonal transport	- Non-invasive	- Larger sample clinical trials necessary to confirm efficacy in humans
	- May work synergistically with trophic factors to rescue RGCs	- Positive results in animal and human small sample trials	
*Stem cell transplantation*	- Direct stem cell replacement of diseased RGCs	- Neuroprotective potential for surviving RGCs	- Expensive
	- Mesenchymal stem cell transplantation promotes survival of RGCs through neurotrophic factors, growth factors, and other neuroprotective cytokines	- Neuroregenerative potential for degenerated RGCs	- Invasive
	- Stem cell replacement of trabecular meshwork cells improves aqueous humor outflow and RGC neuroprotection	- Demonstrated potential for integration with preserved functionality	- Sparse human clinical trials in glaucoma that show equivocal results
			- Challenges in cell purification and protocol
			- Unclear risks and benefits regarding the origin of different stem cell transplant sources
*Exposure to hypoxia*	- Low-dose intermittent hypoxia exposure preconditions neuroprotective cellular responses, increases antioxidant production, promotes hypoxia-inducible factors expression, and protects RGCs against future hypoxic stress	- Strengthens adaptive neuroprotection response that sustains past initial treatment exposure	- Lack of clinical trials in glaucoma
		- Potential for post-injury treatment exposure to have positive effects via adaptive cellular plasticity	- Unclear standardization of recommendations and treatment protocol
*Gene therapy*	- Targeted alteration of a multitude of genes can act by upregulating expression of healthy DNA, proteins, and mitochondria or by downregulating pathogenic mutant forms	- Several genetic associations with POAG have been identified	- Expensive
		- Potential for high-risk loci alteration prior to disease onset or progression	
		- Highly individualized care	- Invasive
	- Genes associated with glaucoma pathogenesis and progression have been identified at various steps throughout the pathway of disease	- Clinical utilization of genetic screening in families	- Early stages of human studies in glaucoma
		- Distinct anatomy of the ocular system conducive to gene therapy	- Improved DNA vector design for effective delivery of genetic material needed
*Mitochondrial transplantation*	- Restores mitochondrial function and cell structure	- Multiple routes of administration possible to achieve desired results	- Expensive
			- Invasive
	- Increases the proportion of healthy mitochondria within a heteroplasmic state	- Successful uptake in human induced pluripotent stem cell-derived RGCs	- Persistent challenges in functional integration and incorporation of mitochondrial material
		- Ability to produce neuroprotective and altering results in brain neural tissue	- Need for more glaucoma-specific studies
*Light therapy*	- Enhances mitochondrial energy production, enzymatic activity, cell signaling, neurobiogenesis, and neuronal growth	- Low cost	- May not be suitable for patients with photosensitivity
		- Accessible	
	- Prevents dendritic pruning and RGC degeneration	- Non-invasive	- Unclear treatment protocol standardization
		- Innate characteristics of ocular and mitochondrial systems conducive to light therapy	
		- Demonstrated results in various ocular pathologies	- Lack of clinical trials

### 2.1 Exercise

Current treatments for mitochondrial diseases focus on reducing ROS production, promoting normal mitochondrial DNA (mtDNA), and increasing the proportion of healthy mitochondria, thereby preventing further damage during periods of physiological stress and pathologic conditions (Parikh et al., 2009; Bhatti et al., 2017). Many previous studies in experimental and clinical settings have demonstrated decreases in mitochondrial mass, alterations in mitochondrial morphology, reductions in oxidative capacity, and decreases in mitophagy (i.e., the ability to remove dysfunctional mitochondria) associated with aging and diseases, including glaucoma (Chistiakov, 2014; Coughlin, 2015). Physical exercise has been shown to promote mitochondrial biogenesis pathways and cellular respiration capacity, increasing healthy mitochondrial mass, structure, and function (Rey, 2022). Exercise has also displayed beneficial effects on mtDNA and its expression (Theilen, 2017). Downregulation of mitochondrial complex I, the first enzymatic protein complex in the mitochondrial OXPHOS pathway, has been suggested to contribute to mitochondrial dysfunction and neurologic diseases (Norat, 2020). Previous experimental studies in mice have shown that exercise addresses this deficiency by promoting mitochondrial complex I activity in the brain through both increased mRNA expression and inhibitor resistance (Aguiar, 2014; Gudson et al., 2017). Published studies involving humans have centered around the effects of exercise on mitochondrial health in skeletal muscle. In concordance with experimental studies in the animal brains, these studies in human skeletal muscle demonstrate advantageous outcomes of exercise through similar mechanisms of improved biogenesis and mitophagy (de Oliveira et al., 2021).

Physical exercise has demonstrated benefit in addressing the pathogenesis and symptoms of a wide variety of diseases, with recommendations for exercise as treatment in at least 26 chronic diseases (Pedersen and Saltin, 2015). These include psychiatric, metabolic, cardiovascular, pulmonary, musculoskeletal, cancer, and other systemic disorders (Pedersen and Saltin, 2015). Exercise has been found to act on multiple pathways to produce beneficial results in these varied diseases. In metabolic disorders (e.g., diabetes mellitus) linked to oxidative stress resulting from chronic hyperglycemia, exercise has been found to decrease disease risk by alleviating ROS overproduction (Oguntibeju, 2019). Exercise has also been shown to be particularly beneficial in neurodegenerative diseases through the promotion of neural mitochondrial activity (Wang and Zheng, 2019). In both rodent models and human trials, physical exercise has been shown to increase neurotrophic factors (e.g., brain-derived neurotrophic factor), which in turn activate cascade pathways that eventually reduce mitochondrial dysfunction and neural excitotoxicity (Gold et al., 2003; Rojas Vega et al., 2006; Lau et al., 2015; Vecchio et al., 2018). These findings have been cited to occur following a range of physical activity, from acute anaerobic to regular aerobic exercise, though meta-analyses suggest that regular aerobic exercise may be more effective in increasing peripheral neurotrophic factor levels, synaptic plasticity, and neurogenesis (Ferris et al., 2007; Rothman et al., 2012; Dinoff et al., 2016). Other beneficial effects believed to be exerted on the brain by exercise include support of surrounding glial cells and maintenance of cerebrovasculature (Vecchio et al., 2018).

Consistent with findings in studies of other neurodegenerative diseases, investigations regarding exercise in glaucoma specifically have also identified favorable outcomes. In experimental mouse models with induced IOP elevation, exercised aged mice had similar retinal and optic nerve function compared to non-exercised young mice, as well as improved inflammatory stress response (Chrysostomou et al., 2014). Aerobic exercise has also proved to be effective in reducing IOP, though the exact mechanism has not yet been determined and requires further study (Yuan et al., 2021). Evidence does show that the IOP-lowering effects of exercise are longer-lasting in people who consistently engage in high intensity activity (Qureshi et al., 1996). In a study comparing ocular parameters before and after aerobic exercise in POAG patients and control participants, researchers noted a decrease in IOP and increase in Schlemm’s Canal (i.e., a structure involved in aqueous humor drainage and IOP reduction) dimensions in both cohorts (Yuan et al., 2021). These findings suggest that physical activity may be useful in preventing age-induced RGC vulnerability and in treating various contributors to POAG pathology.

Although studies linking the effects of exercise to mitochondrial function in glaucoma models are limited, published findings suggest that exercise may potentially address glaucoma pathology through mitochondrial pathways. Decreases in nicotinamide adenine dinucleotide (NAD+), a protective metabolite and important molecule in energy production, occur with age in the mitochondria of both normal and glaucomatous RGCs, with negative impacts on mitochondrial ability to manage oxidative stress and resist damage from high IOP (Williams et al., 2017). Gene therapy and NAD+ precursor (B3) supplementation treatments aimed at inducing NAD+ production have been successful at preventing further RGC and visual function loss, which suggest that NAD+ generation may be a key target for mitochondrial homeostasis and neuroprotection in RGCs (Cimaglia et al., 2020). Exercise may act as a neuroprotective mitochondrial treatment approach for POAG through this pathway, as research has shown that aerobic and resistance exercise training increases the levels of NAD+ in human tissues (Bhatti et al., 2016; de Guia et al., 2019). More research on the effectiveness of exercise in improving mitochondrial output and function in glaucomatous eyes is warranted.

### 2.2 Diet and nutrition

Dietary management of chronic conditions presents an attractive opportunity for more low-cost, accessible treatments as compared to conventional pharmacologic options. Some diets have more conspicuous connections with specific diseases (e.g., low-glycemic diet for type 2 diabetes mellitus, low-sodium diet for chronic kidney disease), while other diets have been suggested to generate an overall health benefit (e.g., Mediterranean diet associated with increased longevity) (Trichopoulou and Vasilopoulou, 2000). The beneficial and deleterious effects of different types of diets in mitochondrial diseases and POAG have been the subject of investigation, with researchers in search of the so-called “mitochondria nutrients” that sustain mitochondrial function (Khalil et al., 2022).

Change in patient diet to treat mitochondrial disease is highly dependent on the targeted disease and the individual patient’s metabolism (Parikh et al., 2009). Ketone-based treatments (ketogenic dietary supplements and ketogenic diet) are a group of diet-based treatments shown to have the potential to improve cognitive function, as demonstrated in studies conducted on neurodegenerative diseases such as mild cognitive impairment (MCI) and Alzheimer’s disease (AD). Ketone-based treatments are thought to improve cognitive health in these diseases through a variety of mechanisms, including attenuation of the abnormal protein accumulation process, reduction of neurotoxicity, improved neurovascular function, and energy rescue in the setting of impaired neuronal glucose utilization (Kashiwaya et al., 2013; Zilberter et al., 2013; Cunnane et al., 2020; Neth et al., 2020). One study found that a ketogenic diet increased optic nerve mitochondrial biogenesis and axonal survival in murine models of glaucoma through the proposed mechanism of increased monocarboxylate transporters that improve substrate availability (Harun-Or-Rashid et al., 2018). Another study in murine models noted a dose-dependent neuroprotective effect of ketone bodies on RGC survival against neurotoxic conditions, suggesting a mechanism of action against neurotoxic metabolites and their downstream effects (Thaler et al., 2010).

Increased fatty acid exposure commonly seen in modern-day dietary habits has also been associated with mitochondrial dysfunction and induction of pro-inflammatory adipocytokines (Khalil et al., 2022). Adoption of a low-fat diet can address a significant source of fatty acid exposure that predisposes to mitochondrial dysfunction and metabolic diseases (Civitarese et al., 2007). Additionally, the Mediterranean diet is another dietary regimen that has been found to increase fatty acid oxidation, mitochondrial biogenesis, and antioxidant capacity through its antioxidant polyphenol-rich foods (e.g., grapes, wine, nuts, legumes, olive oil) (Khalil et al., 2022). Adequate levels of many other micronutrients, vitamins, and minerals (e.g., vitamins B, C, E, selenium, coenzyme Q10, caffeine, carnitine, lipoic acid, *etc.*) are also suggested to contribute to mitochondrial energy metabolism, ATP-production, biogenesis, and molecular metabolism (Wesselink et al., 2019; Fila et al., 2021).

Another approach in dietary treatment of mitochondrial diseases is caloric restriction. Caloric restriction has been demonstrated to reduce ROS production, attenuate age-related oxidative damage, and increase ROS scavenging (Civitarese et al., 2007). Previous studies also demonstrate that caloric restriction increases mitochondrial density, with some proposing increased transcription-induced mitochondrial biogenesis, while others suggest prevention of age-related existing mitochondrial deterioration (Civitarese, 2007; Lanza et al., 2012). Caloric restriction in the form of fasting has been studied in glaucomatous mice models, finding that this method effectively decreases RGC death and has an overall neuroprotective effect (Guo et al., 2016). However, it is essential to note that IOP was not changed in this study, so the neuroprotective effect in this case was unrelated to IOP (Guo et al., 2016). Dietary modifications, such as changes in meal frequency or fasting, require further exploration in patient populations (Parikh et al., 2009).

Iron and zinc are both ion cofactors utilized throughout the nervous system for neurotransmitter biosynthesis, cellular signaling, synaptic modulation, as well as mitochondrial structures and functions (Tang et al., 2021). Dysregulation and typically excessive accumulation of mitochondrial iron and zinc ions (e.g., disruptions in transport, expression, chelation, accumulation, *etc.*) have led to glaucomatous injury to RGCs via mitochondrial structural damage, membrane potential alteration, transportation modification, and other pathways (Tang et al., 2021). Although still early in understanding, dietary modification of iron and zinc ion levels reaching the mitochondria can be considered as a future direction of exploration. Nicotinamide adenine dinucleotide (NAD+) is an integral part of cell respiration, and a decrease in NAD+ content is seen in the aging process linked to the progression of glaucoma (Williams et al., 2017). Nicotinamide (vitamin B3) is an NAD+’s precursor, and previous studies have focused on the effects of vitamin B3 on glaucoma development in mice. Vitamin B3 oral supplements were given to mice before and after IOP elevation, and results displayed reductions in optic nerve degeneration in both groups (Williams et al., 2017). These findings suggest that Vitamin B3 oral supplementation is a possible prophylactic and therapeutic option for glaucoma. Like vitamin B3, other molecules with antioxidant properties are a promising focus of nutrition studies. Oxidative stress in glaucoma patients becomes present as the disease progresses, increasing the importance of antioxidants. Resveratrol, coenzyme Q10, vitamin E, alpha-lipoic acid, omega-3 fatty acids, and hesperidin are suggested antioxidants that could be introduced into the patient’s diet (Dziedziak et al., 2021). Since antioxidant supplementation is a non-invasive and possibly an easily accessible treatment, further study of this subject through clinical trials would be valuable. This topic of antioxidant therapy is further discussed in this paper.

Improvement in the quality and quantity of calorie intake has been suggested to produce multiple benefits in mitochondrial function. A variety of dietary alterations that involve decreasing, increasing, or substituting certain nutrients have been demonstrated to mitigate mitochondrial dysfunction and POAG. Increased studies in patient populations would be necessary to assess efficacy in human populations and real-world conditions, which could demonstrate poorer adherence to strict dietary restrictions and raise concerns for inadequate nutrition balance in vulnerable populations (Włodarek, 2019).

### 2.3 Antioxidant supplementation

The mitochondria constitute a major energy source for cellular functions via the mitochondrial oxidative phosphorylation system. A deleterious byproduct of mitochondrial cellular respiration is the release of ROS, which are a class of free radicals and oxidants. While ROS and other pro-oxidants are necessary for cellular signaling and defense, overaccumulation leads to oxidative stress and damage to both the organelle and the cell (Silwal et al., 2020). Disruptive consequences of excessive oxidative stress include increased membrane permeability, altered calcium regulation, induced mtDNA and nDNA mutations, and resultant cell death (Guo et al., 2013). Of note, the mitochondrial genome primarily encodes for protein products necessary for the mitochondrial cellular respiration system, which assumes approximately 85% of a cell’s oxygen usage (Tezel, 2006). As a result of their close connection in the location, production, and utilization of oxidant-producing mechanisms, mitochondria are particularly vulnerable to the effects of ROS-induced oxidative stress. In the eye, ocular tissues are consistently exposed to natural and artificial lights, which are prominent sources of oxidative damage (Maiuolo et al., 2022). In order to counter the adverse effects of pro-oxidant species, innate antioxidant pathways exist to preserve mitochondrial and cellular conditions. A variety of enzymatic (e.g., superoxide dismutase, catalase, glutathione peroxidase) and nonenzymatic (e.g., glutathione, vitamin C, vitamin E) antioxidants derived from both endogenous and exogenous (e.g., diet) sources work synergistically to maintain oxidative balance (Oyewole and Birch-Machin, 2015; Caceres-Velez et al., 2022). This favorable antioxidant effect is achieved through a variety of described methods, including blocking the formation of free radicals, interrupting key steps in the oxidation chain, scavenging free radicals, reducing reactive pro-oxidants, chelating metal pro-oxidants, and donating electrons to stabilize free radicals (Tan et al., 2018; Ali et al., 2020).

Mitochondrial oxidative stress is found to be contributory to diseases of aging and chronicity, such as diabetes mellitus, cardiovascular disease, chronic kidney disease, osteoporosis and cancer (Leyane et al., 2022). This connection is also particularly noted in neurodegenerative diseases, including Alzheimer’s disease, Parkinson’s disease, amyotrophic lateral sclerosis, and POAG (Chrysostomou et al., 2013; Guo, 2013). Studies have demonstrated significantly lower plasma antioxidant concentrations, higher oxidative stress biomarkers, and more mitochondrial morphologic abnormalities in tissue biopsies of patients with neurodegenerative diseases (Bourdel-Marchasson et al., 2001; Sasaki et al., 2007; Guo et al., 2013; Baierle et al., 2015). ROS-induced oxidative stress leads to neuron damage through glutamate neurotoxicity, which has also been found to apply to RGC’s (Ko et al., 2000; Atlante et al., 2001; Nucci et al., 2005). In RGCs, oxidative stress has direct cytotoxic effects, indirect effects via downstream second messenger signaling and modification, and alteration of surrounding glial cell activity (Tezel, 2006). Studies have noted increased antioxidant enzyme activity throughout the retina in patients with POAG, suggesting a compensation mechanism to higher oxidative stress (Yang et al., 2001; Ferreira et al., 2004). In several experimental models, IOP elevation induced ROS generation and antioxidant depletion in the retina of rodents (Moreno et al., 2004; Ko et al., 2005; Tezel, 2006).

In addition to neuronal RGCs, previous studies also demonstrate the impact of oxidative stress on other cells involved in the disease etiology of POAG. Trabecular meshwork cells facilitate aqueous humor outflow from the anterior chamber, which is necessary to maintain physiologic IOP homeostasis. Dysfunction in these cells leads to increased aqueous humor outflow resistance and elevated IOP, so they are often a target in surgical treatment. Trabecular meshwork cells are more sensitive to oxidative damage (as measured by a biomarker for ROS-induced DNA damage) than other ocular tissues (e.g., cornea, iris), and patients with POAG have higher levels of this biomarker in their trabecular meshwork cells (Izzotti et al., 2003; Izzotti et al., 2009; Wang et al., 2019). Some studies have shown that patients with POAG have decreased antioxidant levels and increased oxidative biomarkers in aqueous humor and blood samples (Ferreira et al., 2004; Sorkhabi et al., 2011; Nucci et al., 2013; Li et al., 2020; Caceres-Velez et al., 2022). Overall antioxidant potential has also been shown to be significantly decreased (64%) in patients with POAG and inversely correlated with IOP levels (Ferreira et al., 2004; Tanito et al., 2015). Evidence of ROS-induced damage is evident at multiple steps throughout the neurodegenerative process of POAG.

Consistent with these findings, antioxidant augmentation has demonstrated potential in the therapeutic management of POAG. Previous studies have proved the ability of antioxidants to attenuate glaucomatous neurodegeneration in several experimental animal studies and clinical human trials. Experimental animal glaucoma models with enhanced antioxidant transcription exhibited decreased proinflammatory cytokines in the retina and optic nerve (Yang et al., 2016). Conversely, mice with genetically altered dysfunctional antioxidant responses displayed decreased RGC survival despite similar IOP elevations as controls (Inman et al., 2013). Bovine trabecular meshwork cells genetically programmed to lack the antioxidant glutathione and subsequently exposed to H_2_O_2_ demonstrated outflow resistance, whereas H_2_O_2_ exposure had no effect in control eyes (Kahn et al., 1983). Antioxidants are also suggested to work synergistically with trophic factors to rescue RGCs more effectively than trophic factors alone (90% vs. 81%) (Ko et al., 2000). Coenzyme Q10 (CoQ10) is an antioxidant cofactor of mitochondrial enzymes that is integrally involved with mitochondrial ROS balance and membrane integrity. CoQ10 administration in rat models with high IOP-induced transient retinal ischemia, reduced glutamate excitotoxicity, and RGC apoptotic death (Nucci et al., 2007; Maiuolo et al., 2022). The potential for antioxidant supplementation to be used as not only prevention but also intervention in POAG neurodegeneration pathology was emphasized in a recent study. In this study, two groups of glaucoma mice models were either raised on an antioxidant-supplemented diet or were later added to the same antioxidant-supplemented diet. Both cohorts demonstrated improved oxidative balance, RGC survival, and axonal transport compared to controls (Inman et al., 2013). This would be a significant direction for research as a possible therapy to reverse neurodegeneration.

Exogenous dietary sources of antioxidant supplementation have also exhibited beneficial effects in RGC and mitochondrial functional and structural retainment (Garcia-Medina et al., 2020). Vitamin B3 supplementation in the diet of glaucoma-induced mice led to increased RGC density and soma size as well as higher mitochondrial density within RGCs (Chou et al., 2020). Resveratrol supplementation in the diet of glaucoma-induced rats and administration in human RGC cultures mitigated RGC apoptotic death, RGC morphologic abnormalities, mitochondrial dysfunction, and ROS generation (Zhang et al., 2018). In a small human clinical trial, Ginkgo biloba extract supplementation in the diet of patients with normal tension glaucoma (NTG), a subtype of POAG, resulted in an improvement in visual fields (Cybulska-Heinrich et al., 2012). In another study in patients with glaucoma, oral antioxidant supplementation showed an increase in antioxidant potential and decrease in oxidative DNA damage in patients with high oxidative stress level (Himori et al., 2021).

Much of what is currently known about antioxidant efficacy in disease treatment has been derived from animal studies. Results of human clinical trials are limited due to the challenge of delivering and concentrating agents into the targeted subcellular mitochondria with specificity (Tan et al., 2018; Zinovkin and Zamyatnin, 2019). There are currently no available antioxidant treatments for application to neurodegenerative diseases and no ongoing human clinical trials in POAG specifically. However, several mitochondria-targeted antioxidants (e.g., MitoQ, SkQ1, SS-31, other quinones) have demonstrated promising results in neurodegenerative disease models and human clinical trials in other ocular diseases (e.g., Leber hereditary optic neuropathy, dry eye syndrome, diabetic macular edema, age-related macular degeneration, Fuchs’ dystrophy) (Zinovkin and Zamyatnin, 2019; Jiang, 2020; Ji and Yeo, 2021). Although researchers are currently in the process of developing effective antioxidant supplementation and augmentation strategies for better evaluation of trial efficacy, early studies in animal models and humans have demonstrated the potential for antioxidant therapies in the prevention and treatment of neurodegenerative diseases such as POAG.

### 2.4 Stem cell transplantation

RGCs, like other neurons, are postmitotic cells that do not undergo further differentiation or division. Due to this, RGCs are unable to regenerate following loss in degenerative diseases such as POAG, and this is one of the major challenges to the development of POAG therapeutics. On the other hand, stem cells are undifferentiated cells capable of regeneration, division, and further differentiation into specific cell types. The advances in stem cell culture, reprogramming, and transplantation techniques presents a novel opportunity for RGC replacement therapy. RGC stem cell transplantation therapy research utilizes retinal progenitor cells, embryonic stem cells, induced pluripotent stem cells, and other source cells to develop RGCs (Zhang et al., 2021). It is possible for stem cells to be first transplanted into a model prior to differentiation into targeted RGCs, however, more commonly, stem cell sources are induced into RGCs *in vitro* prior to transplantation into the subretinal space or the vitreous cavity (Zhang et al., 2021).

Stem cell transplantation therapy that aims for direct replacement of dysfunctional or degenerated cells has been explored in both the treatment of mitochondrial diseases and POAG. Clinical trials are in progress to test the safety of stem cell transplantation in mitochondrial neurogastrointestinal encephalopathy (MNGIE), a disorder causing muscular degeneration in the gastrointestinal tract, malabsorption, and weakness in the eyes and other parts of the body (Russell et al., 2020). Studies conducted thus far in a small number of patients have shown that stem cell transplantation, which replaces deficient thymidine phosphorylase and thus prevents mtDNA damage by excess deoxynucleotides, is a successful method for improving symptoms and survival (Parikh et al., 2009; Bax et al., 2019). Likewise, the potential of stem cell replacement of RGCs presents an avenue for further study. Researchers have successfully differentiated human induced pluripotent stem cells into pure populations of mature RGCs and integrated them into murine retina with preserved functionality (Chavali et al., 2022). Other investigators have utilized human adult periodontal ligament stem cells to develop retinal progenitor and retinal ganglion-like cells that also demonstrate maintained functional status as well as capacity for further differentiation (Huang et al., 2013; Ng et al., 2015).

The effects of transplanting stem cells that are not intended to differentiate into RGCs have also been studied in glaucoma pathology. Intravitreal transplantation of mesenchymal stem cell into the eyes of glaucoma-induced rats resulted in increased survival of RGCs, likely due to stem cell secretion of neurotrophic factors, growth factors, and other neuroprotective cytokines (Yu et al., 2006; Zhang et al., 2021). Mesenchymal stem cells are also capable of transferring healthy mitochondria and cell materials to damaged cells, leading to improved overall mitochondrial function and reduced apoptosis (Zhang et al., 2021). This transfer has been successfully demonstrated in corneal endothelium, retinal pigmented epithelium, and photoreceptors cells, but researchers have noted sparser transfer to cells located in the inner retina such as RGCs (Zou et al., 2019; Jiang D. et al., 2020). Stem cell replenishment of the trabecular meshwork system in experimental glaucoma models has also been shown to act on underlying disease pathologies, with studies demonstrating improvements in IOP levels, aqueous humor outflow, trabecular meshwork cellularity, and RGC neuroprotection (Zhu et al., 2016; Zhu et al., 2017).

Human clinical trials on stem cell treatment of glaucoma are largely absent. One trial involving mesenchymal stem cell transplantation in two eyes with advanced glaucoma showed no changes in visual function or electroretinographic response (Vilela et al., 2021). Although limited in sample size, this study is the only completed human clinical trial of stem cell therapy in glaucoma reported at this time. Only a few other clinical trials with small study sample sizes are published on optic neuropathies in general, which have reported positive results. In a case report of mesenchymal stem cell transplantation into both eyes of a patient with autoimmune optic neuropathy, researchers observed marked improvements in visual function, macular thickness, fast retinal nerve fiber layer thickness, and medication requirements (Weiss et al., 2015). In a study of patients with toxic optic neuropathy, a combined application of mesenchymal stem cell and electromagnetic stimulation resulted in significant improvements in visual acuity and mean fundus perimetry deviation index (Ozmert and Arslan, 2021). In patients with neuromyelitis optica spectrum disorder, stem cell transplantation was associated with improved self-rated disability, neurologic, and health scores. Impactful molecular changes were also reported, including seroconversion and complement system mitigation (Burt et al., 2019).

There are currently many barriers to human transplantation, such as the cell purification process and determination of the best developmental stage for cells to maximize therapeutic outcomes (Zhang et al., 2021). Additionally, the question of whether to use allogeneic donor cells or autologous induced pluripotent stem cells (iPSC) from the patient’s own body remains (Zhang et al., 2021). While autologous cells are less likely to form tumors and are easier to source, cells derived from a glaucoma patient may still be vulnerable to glaucomatous damage due to genetic risk factors that are still present. This issue suggests that using gene editing techniques to first correct mutations that increase RGC susceptibility to damage, such as the aforementioned changes that occur in mitochondria and affect factors such as NAD+ production and membrane structure, could improve the efficacy of RGC transplantation.

While further exploration is needed in this area, these study findings show a possibility for different stem cell transplantations to both replace already degenerated cells and promote neuroprotection of proximal surviving RGCs (Zhang et al., 2021). Such future clinical application of stem cell therapy would be transformative to glaucoma management, particularly in patients with advanced optic nerve degeneration.

### 2.5 Exposure to hypoxia

Hypoxia, the state of low oxygen concentration in cells, can be caused by factors such as low partial pressure of oxygen in the environment and states of vascular ischemia (MacIntyre, 2014). The body is capable of compensating for hypoxic atmospheric conditions, such as those found at high altitude and large depths, through homeostatic adaptations like pulmonary vasoconstriction, hyperventilation, and increased production of 2,3-diphosphoglycerate (MacIntyre, 2014). All of these adjustments provide increased oxygen delivery to at-risk tissues. Given oxygen’s importance in ATP production and therefore cellular function, acute and prolonged hypoxia can be deadly. Studies in RGCs, specifically, have shown that exposure to extended periods of hypoxia (achieved through vascular hypoperfusion, evoked hypoxic states, or hypoxia-mimetic agents) induced ROS and oxidative stress elevation, upregulating pathways to RGC apoptosis (Tezel, 2006; Tulsawani et al., 2010).

However, several published studies in the current literature delineate that low-dose intermittent exposure to hypoxia can be a beneficial therapeutic in the treatment of various conditions (Navarrete-Opazo and Mitchell, 2014). Intermittent hypoxia (IH) refers to protocols with modest (9%–16%) inspired oxygen levels and short, infrequent daily cycles of normoxia and hypoxia (Navarrete-Opazo and Mitchell, 2014). Studies showed that IH built tolerance for and was protection against stressors such as myocardial infarction through multiple mechanisms, including increased antioxidant enzyme expression and the opening of ATP-sensitive K+ channels in the inner mitochondrial membrane of cardiac mitochondria (Kolár et al., 2005; Navarrete-Opazo and Mitchell, 2014).

IH in the treatment of mitochondrial disease is an emerging field of exploration. A recent study in a *Ndufs4* knockdown mouse model of Leigh syndrome revealed that IH led to improvement in brain lesions, neurological behavior, and survival compared to normoxic controls (Parikh et al., 2009; Jain et al., 2016). In contrast, mild hyperoxic conditions were found to decrease survival in these mice (Parikh et al., 2009; Jain et al., 2016). Additionally, this study illustrated that important elements of the hypoxic response pathway such as hypoxia-inducible factors are preserved in diseased cells even in normoxic conditions (Parikh et al., 2009; Jain et al., 2016). Hypoxia-inducible factors (HIF) are transcription factors that regulate cellular response to environmental oxygen levels and these are involved in the induction of non-oxidative, alternative energy-generating processes (Tezel and Wax, 2004). Together, these findings depict a state of oxygen hypersensitivity in mitochondrial dysfunction, suggesting that oxygen metabolism is an important determinant of prognosis in mitochondrial disease and may play a role in its pathogenesis (Jain et al., 2016). This study also found that hypoxia-inducible factor 1-alpha (HIF-1α) levels were not uniform throughout the retinal layers and controlled hypoxia of the entire circulatory system may result in more uniform levels and possibly positive effects on disease progression, as seen in Leigh Syndrome (Jain et al., 2016). The study findings illustrate that reducing oxygen consumption and therefore the demand for OXPHOS may decrease the abundance of ROS and slow disease progression in Leigh syndrome and other mitochondrial diseases.

This hypersensitivity to oxygen levels and metabolism along with its contributory role to disease pathogenesis has also been observed in POAG. Mechanisms for mitochondrial injury related to oxygen consumption, such as deficient complex-I driven cellular respiration seen in POAG lymphoblasts, point toward the potential of POAG treatments targeting respiration and therefore mitochondria (Lee et al., 2012). It has also been shown that in the glaucomatous ONH, HIF-1α is significantly more highly expressed in cases than in controls (Tezel and Wax, 2004). In addition, evidence suggests that alongside decreases in important metabolites protective against oxidative stress like NAD, HIF-1α is induced early in disease development (Williams et al., 2017). This result suggests that ischemia in the glaucomatous ONH and hypoxic stress may be involved in the underlying disease mechanism (Tezel and Wax, 2004).

Despite HIF being an indicator of hypoxic conditions, it is important not to directly associate these cellular responses with pathology. Rather, studies have shown that retinal exposure to multiple brief hypoxic stimuli and corresponding increases in cellular response molecules promotes sustained retinal hypoxia tolerance in a process called “preconditioning” (Tezel, 2006; Zhu et al., 2007; Tulsawani et al., 2010; Gidday et al., 2015). In experimental models of mice preconditioned with hypoxia, levels of HIF-1α were elevated for 1 week and its gene target heme oxygenase-1 was elevated for more than 4 weeks following exposure (Zhu et al., 2007). Studies in RGCs have also demonstrated increased antioxidant peroxiredoxin 6 levels within 24 h of exposure, followed by decline and activation of apoptotic pathways after 48 h of exposure (Tulsawani et al., 2010). This finding of adaptive cellular plasticity has also been investigated following glaucoma onset in a “postconditioning” setting (Gidday et al., 2015). In mice with induced ocular hypertension, repetitive hypoxia exposure was associated with improvements in visual evoked potential testing, visual acuity, RGC survival, and optic nerve axon integrity (Gidday et al., 2015).

Although the time periods tested are not consistent across study types and methodologies, the literature concurs that brief, limited hypoxia exposure may lead to an advantageous adaptive neuroprotection response, whereas prolonged hypoxia instigates cell death. The IH approach to treating Leigh syndrome, glaucoma, and other mitochondrial diseases has not yet been applied to humans, but a few clinical trials to test IH in cardiac and neurological diseases are currently ongoing. The potential for therapeutic benefit of controlled hypoxia to protect mitochondria in glaucomatous eyes is worthy of further study.

### 2.6 Gene therapy

Gene therapy is the targeted alteration of a patient’s genetic material that is aimed towards achieving the desired results in disease management. This process can involve a variety of alterations, including insertion, removal, replacement, repair, and regulation of genetic material (Wirth et al., 2013). Vectors introduced to enact genetic change include viruses, nucleic acids, chemical particles, and other microorganisms (Wirth et al., 2013; Panikker et al., 2022). Viral vectors are more commonly employed than non-viral vectors in trials and applications to introduce exogenous genetic information into target cells (Razi Soofiyani et al., 2013). Commonly employed viruses include lentivirus, adenovirus, and adeno-associated virus (AAV) (Razi Soofiyani et al., 2013). Target cells can be germline or somatic in nature, but current clinical application is centered around somatic cell DNA editing to prevent unknown effects on future progeny (Wirth et al., 2013).

Gene therapy was first approved for a human trial in the United States in 1990 to target genetic defects in adenosine deaminase severe combined immunodeficiency (ADA-SCID) (Wirth et al., 2013). Since then, as advancements in gene understanding and targeting continue; exploration into the utility of gene therapy has become an increasingly emphasized field of research. Researchers have noted the value of gene-based therapies in a variety of genetic diseases, due their advantages of providing precise, direct, and effective approaches. Individualization of healthcare is increasingly emphasized, and gene therapy personalized to each patient’s genome and targeted risk loci is a leader of progress in the era of precision medicine. Currently, gene therapy trials and approved clinical applications are focused around cancer and single-gene defect, monogenic diseases (e.g., acute lymphoblastic leukemia, cerebral adrenoleukodystrophy, aromatic L-amino acid decarboxylase deficiency, hemophilia, spinal muscular atrophy, beta-thalassemia) (Wirth et al., 2013; Shahryari et al., 2019). Yet even in multifactorial diseases, targeting of specific contributory genes has been shown to provide benefit (Hou and Kiss, 2019). Clinical trials involving gene therapy in polygenic diseases with multifactorial etiologies are currently ongoing.

Gene therapy has notable ties with the field of ophthalmology due to several features distinctive to the ocular system. Administration of genetic material can occur either *in vivo*, in which new genetic material is introduced directly to the anatomy, or *ex vivo*, in which cells outside of the body are modified before introduction back to the patient (Wirth et al., 2013). While many internal organs are reached using *in vivo* methods, the eyes provide opportunity for both options due to external accessibility and established intravitreal and subretinal delivery methods (Razi Soofiyani et al., 2013). The ocular system is also one of the rare immune-privileged sites of the body, where damaging inflammatory immune responses are limited in area and severity. This improved immune response in eyes is important to note, as concern for the immunogenicity of foreign vectors has been highlighted by the death of a gene therapy clinical trial patient in 1999 due to a massive immune response following high-dose adenovirus vector administration (Stolberg, 1999; Wirth et al., 2013). Additionally, this same physiologic response restriction of the ocular system concentrates vectors to work in the desired area, leading to a lower required dose of vector needed to produce the desired results (Razi Soofiyani et al., 2013; Panikker et al., 2022). Furthermore, neuronal cells of the eyes, which are the pathologic cells and targets of most gene therapies, are differentiated postmitotic cells that allow for even better retention of vectors (Panikker et al., 2022).

Much progress is already underway in the application of gene therapy to eye diseases, especially in the field of retinal diseases (Gregory-Evans et al., 2012). Multiple gene therapy clinical trials are currently ongoing in ocular dystrophies, including Leber congenital amaurosis (LCA), retinitis pigmentosa (RP), choroideremia, Stargardt disease, retinoschisis, achromatopsia, and age-related macular degeneration (Panikker et al., 2022). The U.S. Food and Drug Administration (FDA) approved a gene therapy product in 2017 that uses an AAV2 vector to target the *RPE65* gene in patients with LCA2 and RP (Panikker et al., 2022).

Gene therapy targeting mitochondrial diseases has additional considerations due to the nature of mitochondrial physiology. Originally extracellular bacteria, mitochondria have a separate double-stranded circular DNA (mtDNA) separate from nuclear DNA (nDNA). Additional differences in mtDNA include separate repair mechanisms, ability to replicate independently from nDNA, and heteroplastic capability (Rong et al., 2021). Both mtDNA and nDNA have been found to contribute to mitochondrial diseases and have been the targets of gene-based therapies. Currently, the only ongoing gene therapy clinical trials in a primary mitochondrial disease is in an ocular disease: Leber’s hereditary optic neuropathy (LHON), which is caused by the mitochondrially-encoded *MT-ND4* gene (Slone and Huang, 2020).

In human genome data, researchers found that single nucleotide polymorphisms (SNPs) in the mitochondrially-encoded genes *MT-ND4* and *MT-CYB*, as well as the mitochondrial genome haplogroup K, were associated with POAG (Lo Faro et al., 2021). In a previous study investigating nuclear-encoded mitochondrial gene-sets and POAG, the authors found that multiple lipid metabolism and carbohydrate metabolism pathway gene-sets were significantly associated with POAG and the NTG subgroup specifically (Khawaja et al., 2016). The published literature describes multiple genetic factors that influence mitochondrial pathways contributory to POAG. Nicotinamide adenine dinucleotide (NAD+), a molecule important to protecting cells from oxidative stress and mitochondrial dysfunction, has been shown to decrease in aging RGCs (Williams et al., 2017). In experimental mouse models of glaucoma, gene editing that increased retinal *Nmnat1* expression, which encodes for a NAD+-producing enzyme, was found to be effective in both prevention and treatment of POAG through protecting RGCs from IOP-induced cellular stress (Williams et al., 2017).

Investigators have also noted correlations between glaucomatous mitochondrial dysfunction and endoplasmic reticulum (ER) stress conditions in which unfolded or misfolded proteins accumulate within the ER and lead to a cascade of unfolded protein response (UPR) (McLaughlin et al., 2022). This stress response leads to increased oxidative stress, mitochondrial stress, and cellular vulnerability (McLaughlin et al., 2022). Researchers found that genetic alterations of ER stress related-chaperones, such as DNAJ proteins, were found to alleviate the ER stress response and enhanced cell survival (McLaughlin et al., 2022). *MYOC* gene produces a trabecular meshwork protein called myocilin, and mutations have been consistently associated with raised IOP and inherited POAG (Sharma and Grover, 2021). Researchers have explored various methods to stabilize the mutant form of the protein, decrease pathologic accumulation within trabecular meshwork cells, alleviate the aggregated protein UPR stress response, and prevent subsequent IOP elevation (Burns et al., 2010; Jain et al., 2017; Sharma and Grover, 2021). These include utilizing select chemical chaperone proteins, clustered regularly interspaced short palindromic repeats (CRISPR)-mediated gene editing, and methods to promote myocilin clearance (Burns et al., 2010; Jain et al., 2017; Sharma and Grover, 2021).

In addition to genetic alteration, epigenetic modification has also been explored in the setting of glaucomatous optic atrophy models. Studies of short-term delta-opioid receptor agonist treatment in animal glaucoma models produced long-term RGC neuroprotective effects, preserving RGC functional integrity and cell count (Husain, 2018). One of the proposed mechanisms for the beneficial effects of delta-opioid agonism is epigenetic modification. Histone deacetylase activity has been positively correlated with glaucoma progression, and delta-opioid agonism was demonstrated to suppress this activity in ocular hypertensive animal models (Zaidi et al., 2020). Modification of the epigenetic pathway has also been researched in non-retinal cells, including restoration of cells comprising the trabecular meshwork and Schlemm’s canal system (Sharif, 2021). The inevitable presence of epigenetic variation inherent to humans, which can influence symptom presentation, disease onset, response to therapy, and other aspects of pathophysiology, is another challenging consideration in human disease research and management (Levin et al., 2022).

Application of gene and epigenetic therapy to glaucoma is still in its early stages of development as researchers continue to uncover the underlying relationships between mitochondrial genetics and POAG. Nevertheless, the suitability of the ocular system to gene therapy, mitochondrial gene therapy trials in the ocular disease LHON, and expansion of gene therapy to target multifactorial diseases are promising signs towards future application of gene therapy to POAG.

### 2.7 Mitochondrial transplantation

Mitochondria are primarily inherited maternally by daughter cells in a vertical fashion; however, the exchange of mitochondria has also been noted to occur across cells and tissues within an individual organism (Gäbelein et al., 2021). Mitochondrial transplantation, also called mitotherapy, refers to transplanting active and functional organelles to targeted cells with mitochondrial dysfunction. Since healthy and active mitochondria are essential for cell survival and regulation, the act of replacing dysfunctional mitochondria with normally functioning counterparts offers a direct approach to eliminating the underlying problem and treating the resulting disease (Nascimento-dos-Santos et al., 2021).

Mitochondrial dysfunction has been linked to many diseases, especially cardiac, neurodegenerative, and other chronic systemic diseases (Roushandeh et al., 2019). This mitochondrial connection to a wide variety of diseases positions mitotherapy to be a currently relevant topic with potentially revolutionary effects in treating these diseases of high prevalence (Roushandeh et al., 2019). Mitotherapy has been studied in relation to multiple systemic diseases, including cardiac, respiratory, hepatic, and neurological disorders (Nascimento-dos-Santos et al., 2021). Studies of the effects of mitochondrial transplantation on neurodegenerative diseases have demonstrated promising results, such as the regrowth of neurites and restoration of membrane potentials in neurons (Chang et al., 2019). Other beneficial outcomes include balanced neuroinflammatory response and improved cerebrovasculature supply (Zhang et al., 2019).

The feasibility and impacts of mitochondrial transplantation have also been investigated in neurodegenerative ocular diseases involving RGCs. In an *in vivo* murine study assessing the effects of mitotherapy in rat retina, healthy mitochondria derived from the liver were intravitreally injected into the optic nerve following nerve crush injury (Nascimento-dos-Santos et al., 2020). The results of mitotherapy showed enhanced oxidative metabolism and electrophysiological response, short-term neuroprotection, and increased axon extension beyond the lesion site (Nascimento-dos-Santos et al., 2020). Another study of mitotherapy in *in vivo* mice retina also demonstrated functional uptake of intravitreally injected isolated mitochondria by RGCs (Rotfogel, 2020). These studies suggest the possibility for injected isolated exogenous mitochondria to maintain normal structure and function in *in vivo* RGCs. Additionally, the potential of mitotherapy has also been demonstrated in human *in vitro* studies. A study evaluating iPSC-derived RGCs generated from human Leber hereditary optic neuropathy (LHON) fibroblasts with experimentally corrected mtDNA exhibited restoration of a regular rate of apoptosis and lower levels of mitochondrial oxidative stress (Wong et al., 2017). Another study evaluating the outcomes of mitochondrial transplantation to human skeletal muscle cell-generated iPSC-derived RGCs following induced oxidative damage demonstrated recovery of ATP production and oxygen utilization (Vrathasha et al., 2022). Results of these studies show the ability for mitochondrial alteration to reverse key aspects of disease in human cell-generated iPSC-derived RGC models.

Different logistical parameters, such as the origin of the transplanted mitochondria and its delivery route, affect the ultimate transplantation results (Chang et al., 2019). Currently, two regularly utilized routes of administration for mitochondrial transplantation exist: direct (or proximal) and systemic (Nascimento-dos-Santos et al., 2021). In the systemic route, administration of mitochondria through intravascular injection allows more area to be covered and mitochondria to be distributed more evenly (Nascimento-dos-Santos et al., 2021). However, if a more concentrated delivery of mitochondria is the goal, direct injection to the target site is a more efficient method (Norat et al., 2020). Of note, both direct and systemic routes of administration have produced desired results in murine brains, exhibiting the capability of mitotherapy to overcome the common challenge of crossing the highly selective blood-brain barrier to produce results in brain neural tissue (Shi et al., 2017). Development of novel mechanisms of cell-to-cell transplantation of mitochondria (and other macromolecules and organelles) are currently ongoing (e.g., FluidFM-based, cybrid technique) (Wong et al., 2017; Gäbelein et al., 2021).

Published studies support the potential for mitochondrial transplantation to be an effective method of reversing debilitating glaucomatous damage caused by mitochondrial pathologies. The current literature contains studies and evidence pointing to the effectiveness of mitochondria transplantation in different animal and cell models across neurodegenerative and ocular diseases. However, a need for more literature investigating mitochondrial transplantation in studies pertinent to POAG is evident.

### 2.8 Light therapy

Light therapy, also called phototherapy, refers to the specific exposure of tissue to artificially generated light energy in the treatment of various conditions. Perhaps the most widely known medical application today is in the field of psychiatric disorders as an intuitive counter to seasonal affective depression (Rosenthal et al., 1984). The impact of light therapy on the human condition has been demonstrated throughout studies in different scientific fields. Meta-analyses of randomized control trials have revealed significantly decreased depression symptoms and significantly increased efficacy of light therapy compared to placebo in seasonal affective disorder and nonseasonal depression (Golden et al., 2005; Pjrek et al., 2019). Other common utilizations of light therapy that are well-known to both the scientific and general communities today include its dermatologic applications. Phototherapy has established uses in diseases with dermatologic involvement (e.g., neonatal jaundice, psoriasis, vitiligo, eczema), as well as dermatologic features of cosmetic concern (e.g., wrinkles, texture, pigmentation) (Ablon, 2018; Rathod et al., 2022).

Researchers have noted that different light exposures (e.g., time of day, length of exposure, wavelength of light, amplitude of light, duration of use) can be designed to address different types of disease (Rathod et al., 2022). For example, bright white phototherapy is heavily utilized in mood disorders in contrast to light emitting diode (LED) phototherapy that is utilized in dermatologic diseases (Golden et al., 2005; Ablon, 2018). One subset of light therapy includes low-level laser therapy (LLLT), also called photobiomodulation therapy (PBMT). LLLT is so-named as it applies red to near-infrared (NIR) light energy at lower densities to tissues (Chung et al., 2012; Hamblin, 2018). Following the invention of the first lasers circa 1960, LLLT quickly emerged as a possible therapeutic modality and research subject of interest when a ruby laser was demonstrated to promote wound healing and hair growth in mice (Chung et al., 2012). Due to its lower energy densities, LLLT also has noted benefit in preventing unwanted increases in the temperature of recipient tissues (Chung et al., 2012; Hamblin, 2018). No significant side effects have been reported with LLLT in the studied literature, making it a non-invasive and non-toxic procedure suitable for further studies (Osborne et al., 2016).

LLLT is particularly applicable to mitochondria and eyes due to several features distinct to these systems. Mitochondria are the primary locations for light absorption throughout human tissues due to many mitochondrial proteins containing compounds (e.g., chromophores, porphyrin, flavins) programmed for light absorption (Osborne et al., 2017; Hamblin, 2018). Additionally, the mitochondrial electron transport chain has been found to be particularly photosensitive to LLLT due to its terminal enzyme cytochrome C oxidase (Tafur and Mills, 2008). Cytochrome C oxidase has been identified as the primary photoreceptor of LLLT, and it is particularly important for oxidative metabolism in the eyes and neurons (Tafur and Mills, 2008; Zhu et al., 2021). Studies have shown that NIR light delivery significantly increases cytochrome c oxidase production, activity, and reduction in neurons (Eells et al., 2004; Zhu et al., 2021). In addition to increasing electron transport chain enzymatic activity, LLLT has also been associated with increased production of ATP in mitochondria (Karu et al., 1995). Studies that demonstrated LLLT-induced increases in ROS to levels conducive to enhanced cell signaling observed promotion of several transcription factors and genes involved in cellular biogenesis, growth, and signaling (Chung et al., 2012). In regard to the ocular system’s relation with light, retinal tissue is known to be the only tissue in the central nervous tissue that receives light exposure (Osborne et al., 2017). The retina absorbs light in the wavelength range of 390–1,000 nm, which encompasses red to infrared light (630–1,000 nm) in the visible light spectrum (Osborne et al., 2017). In consideration of what is presently known regarding the impacts of light therapy on the innate biology, it is vital to study the effects of LLLT on mitochondrial and ocular diseases (Hamblin, 2018).

LLLT has been explored in the treatment of a variety of conditions, such as inflammation reduction, wound and skin ulcer healing, spinal cord injury axonal regrowth, and nerve repair (Zhu et al., 2021). A study of mice hippocampus using 660 nm light showed an increase of brain-derived neurotrophic factor (BDNF) and inhibition of cell death due to a decrease in oxidative stress (Heo et al., 2019). Further studies showed NIR light therapy to benefit retinal pathologies, including prevention of RGC degeneration by correcting mitochondrial dysfunction (Zhu et al., 2021). In addition, 670 nm light has displayed neuroprotective effects against toxins such as cyanide and excessive light-induced damage (Albarracin et al., 2011; Zhu et al., 2021). Dendritic pruning has been linked to glaucoma, and it has been suggested that damage to dendritic cells occurs prior to RGC death in experimental cases (Beirne et al., 2015). Evidence shows that if the retina is treated early after optic nerve axotomy with 670 nm light, dendritic pruning could be prevented in the following hours (Beirne et al., 2016). Other benefits of LLLT include the mitigation of oxygen-induced degeneration, amelioration of lesions in diabetic retinopathy, and attenuation of histopathological changes in animal retinas *in situ* (Osborne et al., 2017). In studies of patients with retinal diseases, LLLT was associated with improved drusen volume and visual function in patients with dry age-related macular degeneration, as well as improvements in retinal thickening and visual acuity in patients with diabetic macular edema (Tang et al., 2014; Eells et al., 2017; Merry et al., 2017). A human clinical trial comparing LLLT to routine IOP-lowering pharmacology in patients with glaucoma is currently listed with no participants recruited to date (He, 2022).

Research regarding LLLT in POAG is limited, but currently light therapy appears to be a promising option as a non-invasive treatment approach with particular impacts on the structures of interest. Further research on LLLT in POAG is recommended to clarify underlying mechanisms and potential impact.

## 3 Discussion

Mitochondrial involvement in disease has become a topic of heightened scientific interest in recent years. Studies reporting the profound impact of the mitochondria on cellular processes have fueled growing research inquiries, which now cover a wide range of diseases and mechanisms, including glaucoma (Murphy and Hartley. 2018). The role of mitochondria in glaucoma pathogenesis has been explored in a variety of animal and human studies, revealing both pathology inducing and ameliorating outcomes that coincide with different mitochondrial alterations (Williams et al., 2017). Although many steps still need to be taken prior to clinical application in patients, published studies in the current literature suggest the possibility of future utilization of mitochondrial therapeutics as treatment modalities in glaucoma. In the present review, we discussed the key mitochondrial therapies with proposed relevance to POAG, highlighting the background, published studies, and limitations or future directions of each approach.

The published literature has identified mitochondrial dysfunction as a contributory factor involved in the pathogenesis of glaucoma (Murphy and Hartley, 2018). Proposed mechanisms demonstrated in previous studies include increased oxidative stress, mtDNA and nDNA genetic mutations, age-related vulnerability, pathway dysfunction, and signaling dysregulation (Chrysostomou et al., 2013; Williams et al., 2017; Mancino et al., 2018). Mitochondrial therapeutics have been found to address key steps in each of these underlying pathologies, and many therapies overlap in areas of influence and action. One of the most common therapeutic targets reported in the described therapies is mitigation of ROS overaccumulation and oxidative stress through various mechanisms of reducing ROS production, promoting antioxidant capacity, and increasing ROS scavenging (Civitarese et al., 2007; Tulsawani et al., 2010; Williams et al., 2017; Heo et al., 2019; Oguntibeju, 2019; Nascimento-dos-Santos et al., 2020; Himori et al., 2021). As retinal tissues are particularly vulnerable to oxidative stress due to high concentrations of mitochondria and high bioenergetic requirements present physiologically, treatment options targeted towards this etiology are especially relevant and beneficial to POAG (Barron et al., 2004). Another common target of therapy includes increasing cellular and mitochondrial biogenesis or supply of healthy mitochondria, which can improve normal heteroplasmic proportions by increasing transcriptional expression, growth factors, or direct transfer (Chung et al., 2012; Harun-Or-Rashid et al., 2018; Rotfogel, 2020; Zhang et al., 2021; Rey et al., 2022). Several mitochondria targeted therapeutics have also demonstrated advantageous effects on IOP, which is POAG’s primary modifiable risk factor. Exercise, antioxidant supplementation, and stem cell transplantation have resulted in reductions in IOP through structural impacts on the trabecular meshwork system that led to increased aqueous humor outflow (Zhu et al., 2017; Wang et al., 2019; Yuan et al., 2021). Additionally, several treatment options were shown to improve mtDNA expression through promotion of mRNA transcription factors and DNA signaling molecules (Gudson et al., 2017; Williams et al., 2017; de Guia et al., 2019). RGC regeneration is the most sought-after outcome in POAG therapy. Studies in stem cell therapy, mitotherapy, and antioxidant therapy have demonstrated the potential for cellular regeneration or reversal of apoptotic pathways (Inman et al., 2013; Wong et al., 2017; Zhang et al., 2021). Ultimately, all described mitochondrial therapeutics demonstrated some degree of benefit in RGC neuroprotection and survival (Thaler et al., 2010; Tulsawani et al., 2010; Inman et al., 2013; Williams et al., 2017; Cimaglia et al., 2020; Nascimento-dos-Santos et al., 2020; Zhang et al., 2021; Zhu et al., 2021).

Mitochondrial studies focused on POAG are still fairly limited across all study types (e.g., basic science research, experimental animal models, human clinical trials). Much of the published research on mitochondrial therapeutics was conducted in diseases with more established mitochondrial connections (e.g., cardiac, musculoskeletal, and neurologic disorders) or cells with more accessible tissues (e.g., human skeletal muscle cells). Although application of mitochondrial therapies in disease have demonstrated promising results in experimental animal models, human trials are particularly lacking. Many barriers exist in human trials, including treatment administration, accurate data collection, and cross-study comparison. As many of these mitochondria-targeted treatment modalities are in the early stages of understanding and development, administration techniques and protocols are not yet standardized to allow for consistent, effective, and specific delivery of treatment. For example, gene therapy is challenged with DNA vector design, mitotherapy is challenged with functional incorporation into target cells, stem cell therapy is challenged with induced differentiation into a specific cell line, and antioxidant therapy is challenged with subcellular delivery into mitochondria (Tan et al., 2018; Zinovkin and Zamyatnin, 2019; Zhang et al., 2021; Di Donfrancesco et al., 2022). Therapies with more straightforward delivery methods also have concerns surrounding study design and treatment administration. For example, diet and nutrition restrictions are difficult to assure and assess in human studies, and light therapy lacks standardized treatment protocols (Włodarek, 2019; Zhu et al., 2021).

## 4 Conclusion

Currently available therapeutics for the treatment of POAG are both limited and ineffective, rendering certain populations (e.g., non-responders to IOP-lowering treatments and those with advanced neurodegeneration) defenseless amidst a significant gap in care (Doucette et al., 2015). This lack of effective treatment highlights the value of and need for better understanding of POAG and its underlying pathophysiology.

As the present literature continues to draw increasing attention to the mitochondrial contribution in POAG, it is time to also consider the possible future therapeutic applications of this knowledge to affected individuals. Future applications of increased mitochondria-targeted knowledge and therapies include preventative neuroprotection, post-degenerative neuron replacement or regeneration, genetic testing, and family screening.

Although not completely comprehensive of all studied mitochondrial therapies (e.g., another suggested mitochondrial therapeutic not mentioned in this review includes pharmacologic drugs), the present review provides an overview to the current state of understanding, research, and application of several key mitochondrial therapies in POAG. Further experiments, studies, and reviews of all categories are recommended to advance the fields of mitochondrial study and POAG therapeutics.
